# Understanding the socio-structural context of high HIV transmission in kasensero fishing community, South Western Uganda

**DOI:** 10.1186/s12889-015-2371-4

**Published:** 2015-10-08

**Authors:** Muhamadi Lubega, Neema Nakyaanjo, Sumaya Nansubuga, Edgar Hiire, Godfrey Kigozi, Gertrude Nakigozi, Tom Lutalo, Fred Nalugoda, David Serwadda, Ronald Gray, Maria Wawer, Caitlin Kennedy, Steven James Reynolds

**Affiliations:** Rakai Health Sciences Program, Old Bukoba road, PO Box 279, Kalisizo, Uganda; Division of Global Health, IHCAR, Department of Public Health Sciences, Karolinsika Institutet, PO Box 1717007, Stockholm, Sweden; School of Graduate Studies and Research Busoga University, PO Box 154, Iganga, Uganda; Division of Intramural Research, National Institute of Allergy and Infectious Diseases, National Institutes of Health, Bethesda, MD USA; Johns Hopkins University, Bloomberg School of Public Health, Baltimore, MD USA

## Abstract

**Background:**

In Kasensero fishing community, home of the first recorded case of HIV in Uganda, HIV transmission remains high with an incidence of 4.3 and 3.1 per 100 person-years in women and men, respectively, and an HIV prevalence of 44 %, reaching up to 74 % among female sex workers. We explored the social contextual factors for the high HIV transmission at Kasensero to inform future policy and preventive interventions.

**Methods:**

We conducted 20 in-depth interviews, including both HIV positive and HIV negative respondents, and 12 focus-group discussions involving a total of 92 respondents from the Kasensero fishing community from April-September 2014. Content analysis was performed to identify recurrent themes.

**Results:**

Our findings suggest that the high HIV transmission in Kasensero is a complex function of eight themes including; positive/negative attitudes about HIV and combination HIV prevention such as the demand for services versus ART/circumcision disinhibition; HIV depository; Multiple partners; Frequent unprotected sex; Clothing; Parental behaviors; Pressure within the sex industry; and Cross generational sex.

**Conclusions:**

The current combination HIV prevention services by the RHSP need to be enhanced with more government involvement including ensuring sustainable supply of ART and circumcision services since they are reportedly highly demanded. Community involvement through the engagement of popular peers could also help in the campaign to change the HIV predisposing culture, misconceptions and risky social norms of the population.

Social Context HIV Transmission Fishing Community.

## Background

Despite national efforts to reduce HIV transmission, HIV remains widespread among fishing communities in Uganda [1]. In Kasensero fishing community (around lake Victoria) south western Uganda, HIV prevalence is 44.3 % (74.5 % among female bar workers), and HIV incidence is ~4.3/100 person-years among women and ~3.1/100 person-years among men [2]. This prevalence is twice as high compared to the average HIV prevalence (22 %) in fishing communities around lake Victoria in Uganda and six times higher compared to the national HIV prevalence of 7.2 % [3, 4]. Like other key populations, fishing communities contribute disproportionately to the national HIV burden and are at greater risk of infection than the general population. They also play a leading role in spreading the infection to the general population [5, 6].

The government of Uganda is providing combination HIV prevention (CHP) (biomedical, behavioral and social-structural services to reduce the new HIV infections in fishing communities and other key population [7]. In the same line, Rakai health sciences program (RHSP) has been providing CHP services to the Kasensero fishing community for over five years now. The persistent high HIV prevalence and incidence in the Kasensero community is, however, still a cause for great concern and a clear contextual understanding of the status quo is required for generation of more enhanced approaches to improving the current CHP services to the community.

Research in settings similar to Kasensero fishing community in Malawi, Tanzania, Zambia, and other sites around lake Victoria basin has shown that contextual vulnerabilities to HIV acquisition (lack of HIV awareness, transactional sex and health system deficiencies among others) are a function of the social and cultural norms, values, networks and structural settings of a particular community ([8–10]. Similar research has also shown that these contextual vulnerabilities also operate in concert with individual’s behaviors and practices to influence the HIV epidemics in those particular settings [9–12]. It would be exigent, however, to generalize or transfer these contextual underpinnings from these studies/settings to Kasesnsero fishing community for two reasons; The HIV prevalence/incidence in Kasensero is almost twice as high as other fishing communities around lake Victoria. Secondly, Kasensero fishing community is the site where the first case of HIV was diagnosed in Uganda and the HIV/ART awareness are presumably high. Based on the social ecological model of behavior, we therefore sought to explore what social-behavioral contexts can individually or in combination explain the very high and persistent HIV prevalence/incidence in Kasensero compared to other fishing sites. The socio-ecological model (SEM) is based on the intertwined relationship between the individual and the environment. The model asserts that individual behavior is not only a function of a person’s individual knowledge, attitude, perceptions among others but also the environment in which he/she lives. The model caters for the different intertwinned levels of an individual’s environment from intrapersonal (biological, psychological), interpersonal (social, cultural, attitudinal), organizational, community, physical environmental and policy outlines or guidelines as centres for behavioral actions or change [13–17].

## Methods

### Study setting and population

Between April and September 2014, we conducted this study at the Kasensero fishing community Rakai district south western Uganda approximately 200 km from the capital city Kampala. The community constitutes a population of approximately 15,000. The majority of the population is mobile and depends directly or indirectly on fishing. Community members include fishermen, boat owners, fish processors, boat builders, fishing gear makers/repairers, retail traders and other casual income earners. Most people reside in temporary structures some occupied by more than one family. There are over 40 lodges (hotels) and 80 bars open 24 h a day throughout the week with young women whose main source of income is commercial sex There is also a large fish factory which processes and freezes fish and there is heavy traffic of freezer trucks which convey the frozen catch for export to Europe. All these groups have different contextual patterns of interactions, access to money and patterns of seasonal migration and engagement in sexual relationships that might affect their vulnerability to HIV acquisition. The majority of the adult population is not permanently married but has open and short term sexual relationships. Most sexual interactions entail a variety of financial relationships linked with the fishing industry and cuts across all age categories with a lot of cross generational sex. Kasensero landing site is also a social hub for singers from Kampala who are often accompanied by sex workers to attract clientele. Other social events such as film shows/drama groups are held almost daily.

The Kasensero fishing community is part of the Rakai Community Cohort (RCC). The RCC is a prospective cohort in Rakai that is surveyed every 12–15 months by the RHSP for demographic, HIV and reproductive data as well as other communicable and non communicable individual/family health indicators.

The community is served by two outpatient health facilities, one constructed and supported by the Rakai health sciences program (RHSP) and the other by government. Both facilities offer HIV testing and counseling (HTC), pre-antiretroviral (pre-ARV) care, and CD4 monitoring as well as antiretroviral therapy (ART) services every day from Monday to Friday and circumcision services through referrals to the main RHSP thaetre or through field outreach camps by the RHSP to kasensero at least once a quarter. There are ten drug shops/private clinics which are not officially accredited to offer ART but often sell cotrimoxazole, condoms and contraceptives all of which are important components of combination HIV prevention (DHO Un published).

### Data collection tools and methods

This qualitative study employed 12 focus group discussions (FGDs) and 20 in depth interviews (IDIs). Participants were HIV infected and uninfected persons 15 years and older from the Kasensero community (Tables 1 and 2). The FGD and IDI participants were selected from a sampling frame designed to elicit different viewpoints [18]. The participants were chosen from all consenting members of the Rakai Community Cohort (RCC) through maximum variation sampling to capture differences of opinion with regard to age, gender, occupation and HIV transmission at Kasensero. The participants were also selected because they were considered to be more “knowledge rich” about the study topic [18]. Participants were invited to take part in only one FGD or IDI. Each FGD consisted of a maximum of 12 participants. The FGDs were stratified by gender and HIV status. Participants were not told how their groups were selected or their serostatus to avoid potential stigmatization and “playing to type”. Having the information that people previously provided through the RCC surveys enabled us to choose the participants by characteristics such as occupation and HIV status.

**Table 1:** General characteristics of in depth interviewees (IDIs) (*n* = 20)

Variable	Category	No of participants	Percentage (%)
Gender	Male	10	50
	Female	10	50
Marital status	Married	13	65
	Single	07	35
Mean age	29.7		
Age range	15 – 24	08	40
	25 – 34	03	15
	35 – 44	08	40
	45 - 54	01	05
Education	None	04	20
	Primary	11	55
	Secondary	05	25
Religion	Roman Catholic	08	40
	Moslem	05	25
	Protestant	03	15
	Other	04	20
Occupation	Fishing/fish trader	09	45
	Bar owner	02	10
	Student	01	05
	Housewife	03	15
	Motorcycle riding	01	05
	Sex worker	01	05
	Mechanic	01	05
	Tailoring	01	05
	Peasant farming	01	05

**Table 2:** General characteristics of FGD participants (*n* = 92)

Variable	Average characteristic of each FGD	Number	Percentage (%)
Participants Number	Average number per FGD	7.7	
Age	Age range per FGD	15 to 65 years	
Duration	Average duration per FGD	68.2 min	
Sex	Males per FGD	06	50
	Females per FGD	06	50
HIV status mean	Positive per FGD	07	58
	Negative per FGD	05	42

Using a topic guide, we explored individual, network and group attitudes, risky behaviors, perceptions, misconceptions and other social contextual factors that were associated with the high HIV transmission in the area. On average, each interview lasted approximately one hour. Interviews stopped when it was judged that saturation had been reached.

All data collection was supervised and assessed by the first author (ML) who is a Ugandan public health physician experienced in social research for HIV care and the second author (NN) who is a social scientist with experience in qualitative research in the study setting. Five research assistants moderated and took notes for the study. The research assistants were conversant with qualitative data collection methods for over five years, had conducted similar research in the study setting and were fluent in Luganda (the local language). They were trained for two days on the study aim, design and tools. Role-plays were used to prepare the research assistants for their interaction with the informants [19, 20]. The experiences from the role-plays were discussed and further methodological guidance was given. All the FGDs and the IDIs were conducted in Luganda, transcribed and later translated into English by the interviewers. The authors listened to the tapes to confirm the validity of the information. Data collection stopped when information relating to the topic guides revealed no new information.

### Data analysis

Data analysis was iterative including reviews and discussions at different stages of data collection and appropriate modifications were made to the tools to address emerging issues [21, 22]. Content analysis was used to analyze the transcripts. This entailed reading and reviewing texts of the entire interview back and forth to identify meaningful units in relation to the study subject [21–24]. The meaningful units were condensed and coded by categories and themes individually by each of the authors. The authors then discussed their appropriate individual coding until consensus was reached on the appropriate codes and the themes about the study subject [22].

### Ethical clearance

The study was approved by the Uganda Virus Research Institute (UVRI) Science and Ethics Committee (SEC), and the Uganda National Council for Science and Technology (UNCST). We also sought the approval of the Rakai district authorities. As part of the informed consent, the participants were told about the aims of the study, the anticipated benefits and risks, their ability to participate or withdraw at any time, and assured that all information obtained would be kept confidential. For the minors, written informed consent for participation was sought from their guardians or next of kin who signed on their behalf. The participants signed two copies of the consent form before the interview commenced, and one copy was given to the participant.

## Results

Our analysis of textual data generated eight themes including: (1) Service demand versus ART/circumcision disinhibition, (2) HIV depository, (3) Multiple partners, (4) Frequent unprotected sex, (5) Clothing, (6) Parental behaviors (7) Pressure within the sex industry and (8) Cross generational sex. We describe each of these themes in detail below.

### Service demand versus ARV/Circumcision disinhibition

Most informants indicated that the demand for HIV prevention services at Kasensero was high. The informants reiterated that many members of the community sought HIV prevention services including ARVs and circumcision from the RHSP and government facilities at Kasensero because they had been told the services would prevent them from acquiring HIV.

*“Many people here, even the ever busy fishermen have taken up circumcision and even RVs [ARVs] for those who have AIDS because they have been told these services can prevent them from acquiring or transmitting HIV.” (HIV negative female informant, 27 years, bar owner)*

From the accounts of a few informants, the demand for the services was unfortunately associated with a general lack of fear for HIV. Many informants held the view that people in Kasensero did not fear HIV because of the availability of ARVs which had in their view rejuvenated the lives of many of their colleagues from full blown AIDS to normal life. Others even held views that PLHIV who were on ARVs or had been circumcised were not infectious or could not be infected with HIV.

*“The other reason for the high transmission is we no longer fear the disease (HIV). ….. We have also seen friends here who were really badly off [too sick] but started ARVs and are now leaving a normal life. So this makes some people look at HIV just like any other disease such as malaria” (HIV negative, female fish traders FGD)*

*“….those who have been circumcised cannot infect others or even get infected themselves. It is like they have been immunized through the knife”. (HIV positive female informant, 35 years, sex worker)*

### HIV depository

Since in Uganda HIV was first diagnosed at Kasensero as slim disease, there was a general perception across most informants that almost everybody in Kasensero was HIV positive and that no one could avoid becoming infected with HIV given the social and sexual dynamics at the site as cited by the FGD informants.

*“But you know the other thing, Kasensero is a bank for HIV, since it was discovered here every widow or widower who has lost their spouses due to HIV run here because they are acceptable here……I can assure you that everybody who comes here is HIV positive. So really we see not much cause for fearing what we already have”. (HIV positive, 20–34 years FGD)*

### Multiple partners

Multiple partners was one of the reasons given for the high HIV transmission in Kasensesero. From the account of most informants, many people in Kansesero had several concurrent sexual partners depending on financial and social affordability. One man could reportedly have as many women as he could afford in a short period of time as long as he had the money. The women were also readily available to whoever could afford to sustain them, *“…Even the women, are free on market who ever affords takes and keeps one until he is poor. So each man here has about five sex partners like in a month and the women about ten and these are not permanent because they keep changing with who is new in the area and who can afford. So from my view, it is promiscuity that has greatly contributed to the high HIV transmission and HIV prevalence.” (HIV positive male informant, 41 years fisherman).*

### Frequent unprotected sex

Frequent un protected sex was often reported as one of the reasons for the high HIV transmission in Kasensero. Many men reportedly perceived sex with a condom as not being real and not satisfying. They therefore opted for unprotected sex at a little higher cost since the women were willing to give it to them for a little more money as reiterated by the sex workers.

*“A man will give you any amount of money as long as it is unprotected sex. They say they don’t really feel our sweetness when they are using condoms, they actually say it’s not real sex because in reality, the man will be having sex with the condom and not you”. (HIV positive female, sex workers FGD)*

*“…..also no one should lie to you that a woman here in Kasensero, will ever refuse unprotected sex as long as she sees money in the hands. She will refuse initially just to bargain for more but eventually they accept and they also enjoy it” (HIV positive male informant, 19 years)*

### Clothing

As a bait to lure the money loaded fishermen men into sex, the sex workers reportedly dressed in a provocative or seducing way with very short skirts which almost revealed their private sex alluring parts or tight revealing attire. This dressing often enticed men into sex which in turn predisposed them to HIV acquisition as reported, by both sex workers and fishermen themselves.

*“The other trick is timing when they have just come from the lake. Remember they are loaded with money, so we go and put on our miniskirts and seducing perfumes so every man will just be enticed. The moment you enter a bar like this you will certainly get a willing buyer. And these men are funny, he will tell you, for me I have survived the lake, so I want to enjoy you naturally …” (HIV positive female informant, 28 years, sex worker)*

*“These girls put on very short skirts. You look at her brown thighs and immediately feel like doing anything to have sex with her, so you approach her, negotiate and then go for it.” (HIV negative male, 20–34 FGD)*

### Parental behaviors

A few informants attributed the high HIV transmission to what they called “irresponsible parenting”, particularly among the fisher folk and bar owners/barmaids. Some parents reportedly had sex in the presence of their children. Others even sent their young daughters out in the night so as to get space to have sex with their partners which in turn also exposed the daughters to dangerous sexual encounters outside their parents’ houses/shelters. The informats felt that this behavior exposed teenage sons and daughters to HIV.

*“The other big problem are the irresponsible parents. They bring in men or women for sex in their houses whether day or night in the presence of their children, so the children also get the impression that having sex is ok. Some even make funny noises during sex while their children are listening so what do you expect the children to do? They also copy the same behavior. “(HIV positive female, sex workers FGD*)

*“my mother asks me to get out to a friend’s place until she calls me so they can have sex ….. in the process of finding where to stay I also find someone who can look after me for a night and here you don’t look far….. that is how I got into the sex network.…”. (HIV positive female informant, 19 years)*

### Pressure within the sex industry

Some informants attributed the high HIV transmission to risky sex behaviors ascribed to pressures from within the sex industry or an interested third party such as the bar owners. Many barmaids who also worked as sex workers had been brought to Kasensero by the “financially powerful” bar owners. The maids/sexworkers shelter, security and financial support were reportedly offered by the bar owners in exchange for loyalty including who the maids/sex workers had to have sex with protected or un protected which predisposed them to HIV as reiterated by some of the maids themselves.

*“We don’t get paid a salary, ……because she brought you from the village, the boss (bar owner) will instruct you on which of the approaching men you have to go and have sex with. Sometimes she tells you so and so has given us 200,000 UG shillings (76USD) so we shall divide equally, so I allow you to leave work now and go and have sex with him and you just have to accept or she (the bar owner) will fire you.”. (HIV positive female informant, 35 years, bar maid)*

### Cross generational sex

Cross generation sex involving young girls and adult males and occasionally young men with older women in search for social, material support and other benefits was also often reported as a reason for the high HIV transmission. From the account of the informants, due to the nature and context of the sexual encounters in such relationships, normally the young beneficiaries were often compromised for choice, will and ability to practice safe sex or stick to one partner.

*“You know all these young girls and boys are looking for money and they have little choice on nature of sex because they have to survive…. Old men can have sex with little girls because of money and then these young girls subsequently have sex with their age mates and vise-versa. The same boys will again have sex with others etc.…….finally your child and the other ones in the community all acquire HIV from the same old man” (HIV positive, female fish traders FGD).*

## Discussion

Our findings suggest that the high HIV transmission in Kasensero is a complex function of positive/negative attitudes about HIV and CHP such as the demand for services versus ART/circumcision disinhibition and everyone in the community being potentially HIV positive. Multiple partners, frequent unprotected sex, clothing, parental behaviors, pressures within the sex industry and cross generation sex also contribute to the high HIV transmission in Kasensero.

There was a reported demand for circumcision and ART by the Kasesnsero community which fortunately are readily available at the site. The positive attitude or beliefs by the community in these services as a measure for CHP could be explained by the counselling given at the clinics and their community outreaches. It could also have been due to the frequent RHSP outreaches at the site and the recent demand generation activities promoting demedicalized CHP (the “stylish living” campaign). This finding is, however, contrary to other studies in Africa where fishing communities are faced with inaccessibility to care and poor demand for services [25–28]. The uptake and demand for the services is, however, complicated by the often (FGDs and IDIs) reported behavioral disinhibition related to ART and circumcision in the Kasensero community. The disinhibition could be explained by a lack of accurate HIV knowledge, social conventions or impulsivity. It could also have been due to risk compensation under which the people of Kasensero typically adjusted their behavior in response to the perceived reduced level of risk of acquiring HIV due to the presence of ART/circumcision. This finding is however contrary to other population based trials and/or meta-analyses in Rakai and other communities where ART availability and/or circumcision have not actually been found to cause changes into HIV risk behavior such as having unprotected sex [29–33]. The potential for disinhibition notwithstanding, it is important that the community demanded and took up circumcision and ART services as measures for HIV prevention. The readily available ART and circumcision services by the RHSP/government units at community bodes well especially at this time when global efforts are driven towards increased CHP for key populations [34]. The only challenge is how the access and availability of these services at the site can be sustained over time and extended to other nearby collaborating/interacting fishing communities while at the same time sensitizing the community against possible disinhibition. In terms of the social ecological model (SEM), this phenomenon clearly explains how individual, network or community perceptions or misconceptions about the importance of ART/circumcision affected their positive demand for the servies and yet still made them take up risky sexual behaviors.

Although some informants reiterated the uptake of ART and circumcision as HIV preventive measures, others thought they would inevitably acquire HIV because in their view, everyone in Kasensero was either HIV infected or had a very high risk of getting infected with HIV. This negative perception or attitude is likely to engender a sense of helplessness or lack of control to HIV preventive measures. The attitudes or misconceptions about HIV and CHP in these fishing communities (SEM) could be attributed to low levels of education, low social status and marginalization that has been described in similar resource poor settings [35–37]. The misconceptions about CHP methods or the inevitability of acquiring HIV has been reported among other key populations in Kenya and other resource poor settings [38–41].

Further, members in the Kasensero community had multiple extramarital sexual relationships a phenomenon that was socially acceptable. This predisposed them to several sexual encounters in a high HIV prevalence sexual network. Multiple sex partners have been found to increase potential for HIV acquisition or transmission by other studies in other fishing communities [3, 35, 42, 43].

Failure to use condoms in Kasensero could be attributed to the need for money that deprives women of the social bargaining power for safe protected sex and the relatively higher cost of unprotected commercial sex which makes it tempting for the economically vulnerable women to the young men with disposable income (SEM). It could also be attributed to frequent alcohol use and drug abuse that impair insight and judgment and facilitates behavioral disinhibition which are common in redundant fishing communities [44]. Other studies around the Lake Victoria basin have similarly found that the high HIV prevalence and transmission in fishing communities is attributed to the high rates of unprotected sex [35, 42, 45–48].

Short skirts and tight figure revealing clothing by sex workers or barmaids were used to attract the young fishermen for sex. This bait as the sex workers called it worked well especially to the redundant and money loaded fishermen who most likely have spent many days on the lake and are sexually starved. Seductive clothing as a driver for high HIV transmission has also been established in other studies in Africa and the middle east [49, 50].

The actions of some parents in Kasesero also exposed young girls to early sex. Many of these girls either heard or saw their parents have sex with casual partners while other parents sent the girls out at night so they could have sex in the houses with their casual sex partners. Sending the young girls out at night predisposed them to waiting men with money and subsequently to HIV. The effect of predisposing parental behaviors on HIV transmission has also been identified by UNAIDS and other studies in Africa [14, 39, 51–55].

The shelter, security and financial support for the bar maids were offered by the bar owners in exchange for loyalty. The bosses therefore decided which man the sex workers would have sex with, and how much they would charge for protected or unprotected sex. Because of their financial vulnerability, the sex workers lacked the social capital, security and bargaining power for safer sex or even the choice of who to have sex with regardless of the men’s serostatus (SEM). Coercive working conditions have similarly been identified as drivers of high HIV transmission in several studies in Africa and Asia [56–58].

Related to this pressure within the sex industry was the cross generation sex involving young girls and adult males and occasionally young men with older women in search for social, material support and other benefits. Due to the nature and context of the sexual encounters in such relationships, normally the young beneficiaries are likely to be compromised for choice, will and ability to practice safe sex or stick to one partner. Cross generation sex has also similarly been identified as a driver for high HIV transmission in resource poor settings [59–62].

### Study limitations

This study utilized in-depth interviews and focus groups. We did not conduct direct observation of bars or other entertainment venues where sex work frequently occurs. Such observations could have provided additional contextual perspective of what actually happens in these sexual engagements. However, the openness of our informants strengthens our confidence in the findings. We also triangulated our data collection methods (FGDs, IDIs). This helped us to check for consistency and contradictions inside and across the groups and interviewees. The multidisciplinary and native research team was useful in understanding the contextual aspects relating to high HIV transmission in Kasensero from the perspective of the community itself. We feel that the content analysis employed for this study has achieved appropriate in-depth analysis for the purpose of the study.

## Conclusions/Recommendations

The current CHP services by the RHSP need to be enhanced with more government involvement including ensuring sustainable supply of ART and circumcision services since they are reportedly highly demanded. Community involvement through the engagement of popular peers could also help in the campaign to change the HIV predisposing culture, misconceptions, risky social norms and behaviors of the population. Routine counselling about the effectiveness of the services against HIV transmission, however, needs to be done to avoid complacency and the disinhibition that could be associated with ART and circumcision.
